# Complete Genome Sequence of a Lineage IV Peste des Petits Ruminants Virus from Turkey, 2018

**DOI:** 10.1128/MRA.01446-19

**Published:** 2020-04-09

**Authors:** Sabri Hacıoğlu, Simon King, Şirin Gülsün Çizmeci, Öznur Yeşil, John Flannery, Michael D. Baron, Carrie Batten, Paulina Z. Rajko-Nenow

**Affiliations:** a Etlik Veterinary Control Central Research Institute, Virological Research and Diagnostic Laboratory, Ankara, Turkey; b The Pirbright Institute, Woking, Surrey, United Kingdom; KU Leuven

## Abstract

We report the whole-genome sequence of a peste des petits ruminants virus (PPRV) from a lamb exhibiting clinical signs in Turkey in September 2018. The genome of PPRV/Turkey/Central_Anatolia/2018 shows the highest nucleotide sequence identity (97.63%) to PPRV isolated in Turkey in 2000.

## ANNOUNCEMENT

Peste des petits ruminants (PPR) is a highly contagious viral respiratory disease of goats and sheep characterized by high mortality and morbidity. It is an economically extremely important disease of small ruminants, affecting the lives of over 330 million low-income livestock keepers in Africa, the Middle East, and Asia (1). For this reason, PPR is being targeted by the Food and Agriculture Organization (FAO) and the World Organisation for Animal Health (OIE) for global eradication (1). PPR virus (PPRV) belongs to the *Small ruminant morbillivirus* species within the *Morbillivirus* genus of the family *Paramyxoviridae* (2) and has a single serotype but four genetically distinct lineages (3). Lineages I, II, and III are restricted primarily to the African continent, while lineage IV is found throughout Asia and the Middle East and has recently been found in North Africa and, since 2008, in sub-Saharan Africa (4).

The first PPR outbreak in Turkey was officially reported in 1999 (http://web.oie.int/hs2/zi_pays.asp?c_pays=190&annee=1999) and was caused by a lineage IV virus (5), as were all subsequent outbreaks in that country. Since it lies at the junction of Europe and Asia and has a large population of small ruminants, Turkey may be important in the spread of PPRV into Europe. Recently, two PPR outbreaks were reported in the neighboring countries of Georgia in 2016 (6) and Bulgaria in 2018 (7). However, in neither case has the origin of the disease incursion been identified.

In September 2018, clinical signs including respiratory distress, diarrhea, and lesions in the tongue and gingiva were observed in an unvaccinated lamb in Eskisehir, Central Anatolia, Turkey. Total RNA was extracted from 100 μl of lung tissue on the Kingfisher Flex automated extraction platform (Thermo Fisher Scientific, Paisley, UK) using the MagVet universal nucleic acid extraction kit (Thermo Fisher); this sample tested positive for PPRV using a real-time reverse transcription-PCR (RT-PCR) assay (8). Library preparation was performed using the Trio transcriptome sequencing (RNA-Seq) kit (NuGen, CA), and paired-end read sequencing (2 × 150 bp) was carried out using the Illumina MiSeq platform and version 2 reagents. The raw data were quality trimmed and adapter trimmed using Trim Galore (9); subsequently, the 653,988 paired reads were mapped to the reference genome (GenBank accession number AJ849636) using the Burrows-Wheeler Aligner MEM algorithm (BWA-MEM) 0.7.12. Only a short gap in the GC-rich untranslated region between the coding sequences for the M and F proteins was not assembled using the Illumina technology and was later amplified, sequenced, and confirmed using Sanger technology (with the primers GAGGAGAGCCCTATCCCGCG [forward] and GGCGGGTCTCGTTTCCGGTG [reverse]).

The genome of PPRV/Turkey/Central_Anatolia/2018 is 15,948 nucleotides long with a GC content of 47%. Compared with other PPRV genomes, it is most similar to PPR/Turkey/2000 (AJ849636.2) (97.63% identity) and PPRV/India/Izatnagar/1994 (97.20%) (KR140086.1), indicating that this recent PPRV remains very similar to that isolated during the first reported PPR outbreak in Turkey in 1999. In a 322-bp fragment of the F gene, PPRV/Turkey/Central_Anatolia/2018 showed 100% identity with several sequences collected between 2011 and 2017 from Turkey (Konya, Antalya, Afyonkarahisar, Nigde, and Aksaray provinces). Similarly, in another commonly sequenced fragment of the N gene (303 bp), PPRV/Turkey/Central_Anatolia/2018 showed 100% identity with a number of different sequences (collected between 2015 and 2018) originating from the following regions in Turkey: Bursa, Bilecik, Canakkale, Konya, Antalya, Nigde, and Afyonkarahisar. This finding is in line with that of other studies (10), showing an ongoing circulation of PPRV in Turkey.

### Data availability.

The full-genome sequence of PPRV/Turkey/Central_Anatolia/2018 has been deposited in GenBank under the accession number MN657232. The raw sequencing reads have been deposited in the NCBI SRA under BioProject accession number PRJNA599324.

## References

[1] FAO. 2015 Global strategy for the control and eradication of PPR. FAO, Rome, Italy.

[2] Baron MD, Diallo A, Lancelot R, Libeau G 2016 Peste des petits ruminants virus. Adv Virus Res 95:1–42. doi:10.1016/bs.aivir.2016.02.001. 27112279

[3] Banyard AC, Parida S, Batten C, Oura C, Kwiatek O, Libeau G 2010 Global distribution of peste des petits ruminants virus and prospects for improved diagnosis and control. J Gen Virol 91:2885–2897. doi:10.1099/vir.0.025841-0. 20844089

[4] Parida S, Muniraju M, Mahapatra M, Muthuchelvan D, Buczkowski H, Banyard AC 2015 Peste des petits ruminants. Vet Microbiol 181:90–106. doi:10.1016/j.vetmic.2015.08.009. 26443889 PMC4655833

[5] Bailey D, Banyard A, Dash P, Ozkul A, Barrett T 2005 Full genome sequence of peste des petits ruminants virus, a member of the *Morbillivirus* genus. Virus Res 110:119–124. doi:10.1016/j.virusres.2005.01.013. 15845262

[6] Rajko-Nenow PZ, Cunliffe TG, Flannery JT, Ropiak HM, Avaliani L, Donduashvili M, Baron MD, Batten CA 2017 Complete genome sequence of peste des petits ruminants virus from Georgia, 2016. Genome Announc 5:e01091-17. doi:10.1128/genomeA.01091-17. 29025946 PMC5637506

[7] Department for Environment, Food and Rural Affairs, Animal and Plant Health Agency. 2018 Preliminary outbreak assessment for peste de petits ruminants in Bulgaria. Department for Environment, Food and Rural Affairs, London, United Kingdom.

[8] Batten CA, Banyard AC, King DP, Henstock MR, Edwards L, Sanders A, Buczkowski H, Oura CCL, Barrett T 2011 A real time RT-PCR assay for the specific detection of peste des petits ruminants virus. J Virol Methods 171:401–404. doi:10.1016/j.jviromet.2010.11.022. 21126540

[9] Kreuger F 2019 Trim Galore. https://github.com/FelixKrueger/TrimGalore.

[10] Altan E, Parida S, Mahapatra M, Turan N, Yilmaz H 2019 Molecular characterization of peste des petits ruminants viruses in the Marmara region of Turkey. Transbound Emerg Dis 66:865–872. doi:10.1111/tbed.13095. 30525310 PMC7814889

